# Identifying enablers and barriers to individually tailored prescribing: a survey of healthcare professionals in the UK

**DOI:** 10.1186/s12875-017-0705-2

**Published:** 2018-01-15

**Authors:** Joanne Reeve, Nicky Britten, Richard Byng, Jo Fleming, Janet Heaton, Janet Krska

**Affiliations:** 1 Hull York Medical School, Hull University, Cottingham Road, HU67RX, Hull, England; 2 University of Exeter Medical School, St Luke’s Campus, Heavitree Road, EX1 2LU Exeter, England; 3 Clinical Trials and Health Research, Peninsula Schools of Medicine and Dentistry, ITTC, Drake Circus, PL4 8AA Plymouth, England; 4 Warwick Primary Care, Warwick Medical School, University of Warwick, CV4 7AL Coventry, England; 5 Division of Health Research, University of the Highlands and Islands, 10 Inverness Campus, IV2 5NA Inverness, Scotland; 6 Medway School of Pharmacy, The Universities of Greenwich and Kent at Medway, Anson Building, Central Avenue, Chatham Maritime, Chatham Kent, ME4 4TB Canada

**Keywords:** Polypharmacy, Medicines optimisation, Individually tailored care

## Abstract

**Background:**

Many people now take multiple medications on a long-term basis to manage health conditions. Optimising the benefit of such polypharmacy requires tailoring of medicines use to the needs and circumstances of individuals. However, professionals report barriers to achieving this in practice. In this study, we examined health professionals’ perceptions of enablers and barriers to delivering individually tailored prescribing.

**Methods:**

Normalisation Process Theory (NPT) informed an on-line survey of health professionals’ views of enablers and barriers to implementation of Individually Tailored Prescribing (ITP) of medicines. Links to the survey were sent out through known professional networks using a convenience/snowball sampling approach. Survey questions sought to identify perceptions of supports/barriers for ITP within the four domains of work described by NPT: sense making, engagement, action and monitoring. Analysis followed the framework approach developed in our previous work.

**Results:**

Four hundred and nineteen responses were included in the final analysis (67.3% female, 32.7% male; 52.7% nurse prescribers, 19.8% pharmacists and 21.8% GPs). Almost half (44.9%) were experienced practitioners (16+ years in practice); around one third reported already routinely offering ITP to their patients. GPs were the group least likely to recognise this as consistent usual practice. Findings revealed general support for the principles of ITP but significant variation and inconsistency in understanding and implementation in practice. Our findings reveal four key implications for practice: the need to raise understanding of ITP as a legitimate part of professional practice; to prioritise the work of ITP within the range of individual professional activity; to improve the consistency of training and support for interpretive practice; and to review the impact of formal and informal monitoring processes on practice.

**Conclusion:**

The findings will inform the ongoing development of our new complex intervention (PRIME Prescribing) to support the individual tailoring of medicines needed to address problematic polypharmacy.

**Electronic supplementary material:**

The online version of this article (10.1186/s12875-017-0705-2) contains supplementary material, which is available to authorized users.

## Background

Polypharmacy–the use of multiple medicines in one individual on a long-term basis–is now routine in clinical practice [1]. To optimise the potential benefit of polypharmacy on patient-centred outcomes, we must find ways to support compromise between the views of clinicians and patients when making decisions about medicines use [1, 2].

Growing numbers of patients take multiple medicines in order to optimise management of their (often multiple) disease(s). Polypharmacy can improve patient outcomes [1, 3, 4]. However, long term medication use can also be problematic for patients – burdensome in multiple ways [2, 5–14]. Professional responses have seen the introduction of medicines optimisation strategies, aiming to make it easier for patients to take their medicines as prescribed. But non-concordance is common. For some patients, this is a passive process – the result of being overwhelmed by medication demands that they cannot meet. Others engage in an active resistance to medication use based on thoughtful evaluation of the pros and cons of medicines use [12]. Some people are able to negotiate these decisions with their health care practitioners to reflect the needs demands and priorities of their individual circumstances [2, 6]; but this is by no means universal. Capacity to support individually tailored prescribing – what Denford has described as Mutually Agreed Tailoring (MAT) of medicines [2] - is essential to optimising the use of the medical intervention that is polypharmacy [1, 2].

Previous research has highlighted barriers to individualised (whole person) tailoring of general medical decisions within current managed health systems [15, 16]. These include a lack of resources, skills and training supporting delivery of personalised (generalist) decision making. Professionals describe wanting to deliver individually tailored care, but being unable to do so in practice.

Our team are developing a new model of practice (PRIME prescribing) to support the MAT of medicines use highlighted by Denford’s work [2]. To refine our description of the core elements of our new complex intervention, we wanted to look specifically at current professional perceptions of delivering individually tailored prescribing. Our research question asked: what factors enable or limit health professionals in delivering individually tailored prescribed in every day practice? The results will be used to refine our PRIME prescribing model going in to feasibility testing.

## Method

### Research design

A cross-sectional mixed methods study using an on-line survey instrument informed by Normalisation Process Theory (NPT) [17]. The tool aimed to identify perceptions of enablers and barriers to the integration of individually tailored prescribing into routine medical practice in a convenience sample of health professionals.

### Sample

We sought to contact health professionals (doctors, nurses, pharmacists) potentially dealing with problematic polypharmacy including those actively engaged in prescribing medicines, and those supporting patients who are taking multiple medicines. We used a snowball sampling approach to send invitations through known networks of professional contacts including newsletters to GPs, pharmacists and other primary health care professionals. Networks used included the Society for Academic Primary Care (SAPC), regional networks for the Royal College of GPs (RCGP), RCGP First Five network (recently qualified GPs), known Continuing Professional Development networks, the Prescribing and Research in Medicines Management group, the Royal Pharmaceutical Society, and Local Pharmacy Forums. The survey was publicised in the Pharmacy Research UK Newsletter. We also sent Twitter invitations targeting RCGP, SAPC, and known experts in the field; all links included an invitation to pass details on to individuals’ own networks. Our aim was for maximum reach sampling rather than a statistically representative sample. To support internal consistency, we opted to use data only from UK professionals from the 3 main professional groups–doctors, nurse prescribers and pharmacists.

## Instrument development

Was informed by Normalisation Process Theory (NPT) - see Table 1 - with the tool being adapted by the research team from the NOMAD instrument [18], NPT toolkit [17], and a previous survey instrument used to examine barriers to generalist practice [15]. Questions examined whether participants recognised work within each of the four key domains described by NPT as needing to happen for the practice of individually tailored prescribing to become integrated into every day care. All responses were anonymous, however basic characteristics on type and location of professional practice together with years in practice were collected. The draft survey was road tested with a small sample of GPs and pharmacists to ensure it was useable. The full tool is shown in Additional file 1 and can be viewed at this link https://www.surveymonkey.co.uk/r/7MDY8MF

**Table 1:** Using normalisation process theory to assess individually tailored prescribing

Normalisation Process Theory predicts that for a new intervention to become integrated into usual practice, there needs to be continuous investment by all parties in four areas of work. These include
• Making Sense of the intervention: everyone must understand how the intervention is distinct from other ways of working and why it matters
• Engagement: individuals and collectively people must commit to do the work of the new practice
• Action: people must have the skills and resources to deliver the new way of working
• Monitoring: people must get feedback which reinforces and encourages this way of working
May and colleagues designed a 16 item questionnaire to support the critical examination of these areas of work in assessing implementation and integration of ways of working [17]. The tool has been updated into a more user friendly format in the NOMAD tool [18]. We have previously used the toolkit to examine the enablers and barriers to delivery of expert generalist practice (EGP) in the primary care setting [15]. In this study, we will use the same approach to examine individually tailored prescribing.

Data collection: the survey tool (including an invitation, participant information sheet, consent recording and data collection) was loaded on to Survey Monkey. The survey was open for one month (January–February 2016). No personal identifiable information was collected.

### Analysis

For each theme, our analysis sought to identify evidence of successful work supporting individual tailoring together with problem areas. For the quantitative data, descriptive statistical analysis (absolute numbers, percentages and crosstabs reports) was undertaken by JR with support from SPSS software to describe the characteristics of participants and to compare responses between professional groups and years of practice. Free text responses were analysed by JR and JF using a constant comparative approach [15] to identify enablers and barriers to work in each area. An a priori coding framework was used based on the NPT descriptors (see Table 1) but with researchers remaining alert for new themes emerging. Disagreement was addressed through discussion. Using a framework analysis approach used in our previous work in this area [15], findings were critically reviewed to distil key enablers and barriers to generalist practice in each of the four domains described by NPT [17]. We thus sought to describe key changes needed to support implementation of ITP in everyday practice.

## Results

### Describing our sample

Four hundred and forty-four professionals responded to the survey. To enhance the potential generalisability of our findings, we opted to exclude the small number of responses received from practitioners outside of the UK (*n* = 6), and those from outside the three main professional groups involved in prescribing within UK primary care practice - nurse prescribers, pharmacists or GPs (*n* = 25). Our final sample thus consisted of 419 people: 52.7% of whom were nurse prescribers, 19.8% pharmacists and 21.8% GPs (Table 2). There were more women than men in our sample – partly reflecting the strong nursing representation, as well as known gender bias in survey response [54]. We had responses from people at all career stages - early (17.7%), middle (37.2%) and later career (44.9%) – with respondents tending to be more experienced practitioners. Almost one third of our sample felt they were already routinely offering ITP to their patients. GPs were the group least likely to recognise this as consistent usual practice. However, a further 53.2% of participants described offering ITP some of the time – including 80.4% of GP respondents.

**Table 2:** Summarising the professional experience and location of participants

	Nurse prescriber	GP	Pharmacists	Total
Total number (% of sample)	*n* = 234 (52.7%)	*n* = 97 (21.8%)	*n* = 88 (19.8%)	419 (100%)
Gender				
Male (*n*, %)	31 (13.2%)	49 (50.5%)	54 (61.3%)	134 (32.0%)
Female (*n*, %)	200 (85.5%)	48 (29.5%)	34 (38.7%)	282 (67.3%)
Missing data	3 (1.3%)	0	0	3 (0.7%)
Career stage				
Early career: ≤5 years (*n*, %)	58 (24.8%)	5 (5.1%)	11 (12.5%)	74 (17.7%)
Mid career: 6–15 years (*n*, %)	76 (32.4%)	27 (27.8%)	53 (60.2%)	156 (37.2%)
Later career: 16+ years (*n*, %)	99 (42.3%)	65 (67%)	24 (27.2%)	188 (44.9%)
Missing data	1 (0.4%)	0	0	1 (0.2%)
Location of practice				
England (*n*, %)				400 (95.5%)
Scotland (*n*, %)				6 (1.4%)
Ireland (*n*, %)				4 (0.9%)
Wales (*n*, %)				9 (2.1%)
Missing data				0
Reporting currently providing ITP				
Yes, always (*n*, %)	96 (41.0%)	13 (13.4%)	12 (13.6%)	121 (28.9%)
Yes, sometimes (*n*, %)	96 (41.0%)	42 (43.3%)	78 (88.6%)	216 (51.6%)
No (*n*, %)	28 (12.0%)	17 (17.5%)	5 (5.7%)	50 (11.9%)
Missing data (*n*, %)	14 (6.0%)	16 (16.5%)	2 (2.3%)	32 (14.6%)

Analysis of responses from this group are presented under the four NPT domains used in our survey design.

### Sense making

NPT predicts that for ITP to become an integral part of everyday practice, it must be easy to describe, offer a distinct model of care that is different from other ways of working, with all stakeholders being clear about the aims of practice, expected tasks and value/importance of the work [17]. Analysis of quantitative and free text responses revealed four themes in this domain: that ITP is valued by health care professionals; ITP is valuable; ITP lacks clarity; and the value of ITP is not shared/recognised more widely.

Although only a third of our respondents identified themselves as currently offering ITP, 73.4% of respondents reported wanting to do more (67.1% of nurse respondents, 73.9% of pharmacists and 89.7% of GPs. Most participants felt ITP was a legitimate part of their professional role (68.9% of all respondents; 93.8% of GPs, 69.3% of pharmacists and 60.2% of nurse prescribers). ITP is seen as an integral part of wider professional practice with 356 (85.0%) participants reporting that their colleagues were doing ITP all or some of the time. These data indicating the valued and valuable nature of ITP were supported by the free text responses (Table 3, with further detail given in Table 4).

**Table 3:** Summarising themes from qualitative analysis

Sense making	Theme	Description
	ITP valued by health care professionals	Meeting needs of the individual part of professional identity
	ITP valuable to NHS	Professionals recognised the value of ITP to the NHS
	Clarity on ITP	Prioritising the patient/person as the essence of ITP
	Value of ITP not shared	Organisation values and processes don’t support ITP; some patients don’t understand value of ITP
Engagement	Theme	Description
	Leadership (individual and collective)	Key individual leaders, and collective engagement with ITP
	Levels of engagement	Variable levels of engagement, with desire for more
	Patient engagement	Mobilisation of patient engagement through the media
	Barriers to engagement	Included workload, fragmentation of services, fear, patient resistance
Action	Theme	Description
	Formal training	In generalist practice; within specialist
	Experiential learning – phronesis	Learning from experience, including working with patients and colleagues
	Collective action	Value of peer discussion
	Other supports for action	Including the media
	Partial action	Easier to tailor stopping medicines than starting them
	Barriers	Governance (fear), time, ‘head space’ and practical support
Monitoring	Theme	Description
	Mixed feedback	Both supportive and negative feedback on ITP
	Challenge of feeding back	Hard to quantify benefit
	Challenging the status quo	Hard to ‘go against’ the guideline
	Potential power of feedback	Should be a Key Performance Indicator

**Table 4:** Detailed account of themes from qualitative analysis. Provides a more detailed description of the qualitative data as summarised in Table 3

Sense making	Theme	Subtheme	Descriptions from participants
	ITP valued by health care professional	Defines professional role	“our job starts where the guideline ends” (GP) And not just in managing medicines (Nurse Prescriber)
	ITP valuable to NHS	False economy not to	“could improve care and save money” (GP) “Needs to be developed” (Pharmacist)
		But uncertain	“so long as patient don’t miss out” (GP)
	Clarity on ITP	Prioritising the patient	“advising on the suitability for the patient” (Pharmacist) Principle of personalised medicine
	Value of ITP not shared	By patients	“pts… need to understand prescribing as important as prescribing” (Pharmacist)
		By organisational values	“recognition from the powers that be that this is a good thing to do” (GP)
		By organisational structures	“would be difficult to instigate in practice due to protocol driven practice” (Nurse Prescriber) “needs recognition that this is clever subtle stuff that needs skilled practitioners…not readily done by rote” (GP)
Engagement	Theme	Subtheme	Descriptions
	Leadership	Individuals	Key leaders, influential colleagues, trained colleagues support engagement. “working through examples with trusted colleagues” (pharmacist) “I remember a Protected Learning Time session where a geriatrician talked about the rationale for stopping nearly all the medication” (GP) Independent contractual status for GPs supports engagement
		Collective action	Multidisciplinary team working enhances engagement with ITP
	Levels of engagement	Variable	Engage with idea if not the practice (GP) Pharmacists role to recognise the potential need even if don’t do ourselves
		Desire for more	“want to do more discontinuation of meds” (Nurse Prescriber)
	Patient engagement	Media	Media input in to dangers and harms of medicines can help as it starts a conversation
	Barriers to engagements	Excess workload	“limited by time, caseload and so lack of mental capacity” (GP). Time and complexity mitigate against depth of conversation needed. Stopping meds increases workload – follow up consults
		Fragmentation of care; lack of integration of vision and process	Inefficiency crowding out effort; disparity between primary and secondary perspectives, power and resources; population over individual focus
		Fear	Limits engagement “it’s a fear of making a mistake and the potential consequences” (GP)
		Patient resistance	Patients can be reluctant to change “can be difficult to persuade carers and patients to change meds they’ve been taking for a long time and were told were for life” (Pharmacist) Patient expectations and lack of understanding of greyness of medicines
Action	Theme	Subthemes	Descriptions
	Formal training	GP training	Generalist training; basic principles; knowing the guidelines before you deviate off “this wasn’t taught when I was training” (GP)
		Specialist training	Prescribing (stop-start); working within specialist area easier to do ITC
	Experiential learning–phronesis	Self taught/experience	“experience gained intuition”; (GP) practiced at doing this over a long time
		Learn from patients	“just day-to-day learning from patients” (GP)
		Learn from colleagues	Trusted colleagues and influential figures; shared reflection including on line discussion
	Collective action	Peer discussion	MDT and collaborative action supports ITP (but can inhibit decision making too as need full agreement). Supervision
	Other support	Media	To start the conversation
	Partial action		Easier when stopping meds than starting
	Barriers	Organisational practice – pay for performance	Lack of joined up thinking and communication; monitoring as a barrier
		Time	
		Resource	Qualified and experience staff lacking; resource prioritises opposite approach; imbalance need and supply; peer senior support and continuity of same needed; legal support “resource restriction means prioritise safety and supply” (Pharmacist)
		Mental capacity and complexity	“Limited by time caseload and so lack of mental capacity” (GP); exhaustion “To operate outside ‘recognised prescribing’ requires understanding of the clinical evidence supporting the current guidelines, when there are gaps in that evidence and when it is therefore appropriate to choose a different path. An important variable is the patient wishes and how these should be accommodated” (Pharmacist)
		Practical advice	Practical advice, a framework, training
		Fear	Making and recording defendable decisions; being castigated by others – clinicians, legally, morally; uncertainty re risk “Shared balanced discussions with patients rarely results in a DEFENDABLE decision. If you are way of the mark with clinical decisions then it is probably sensible to share your decision with colleagues” (GP) “Fear of being misunderstood & misinterpreted as undertreatment, apathy, fear of going against guidelines & being medicolegally vulnerable” (GP)
Monitoring	Theme	Subtheme	Discussion
	Mixed feedback	Positive	From patients and colleagues helps confidence, helps staff to prescribe less not more – more PCC “each time I see a positive effect am motivated to do more” (Nurse Prescriber)
		Negative	From colleagues (secondary care) and patients (complaints) “I stopped metformin in a 90-year-old with dementia, daughter complained, made me wary to deprescribe” (GP)
	Challenge of feeding back	Demonstrating impact	Hard to quantify benefits (GP)
	Challenging the status quo	Fear of feedback	“If there is a problem may be hard if against the guideline” (GP)
		Monitoring as a barrier	Accept only small deviation, monitoring from population not individual perspective, pressure to prescribe to QOF. “should be a KPI” [KPI = Key Performance indicator]
	Potential power of feedback		Should be a KPI

However, some uncertainties were expressed by professionals about the value and purpose of ITP. For example, one GP valued individual tailoring “so long as the patient doesn’t miss out”. Inconsistencies in responses suggested that some had understood our description of ITP in a different sense than we had intended. We understand ITP to support the potential need for compromise between the preferences and priorities of guideline ‘best practice’ and patient perspectives. Yet we identified examples of practitioners describing that they always work within guidelines, whilst also reporting that they were delivering Individually Tailored Care. Findings also suggested a possible gap between what professionals value/want to do, and how they actually practice. A view echoed in this response from a GP who described:

“I suspect I tailor medicines much less than I would like to think I do” (GP)

Professionals commonly reported that they thought their organisation didn’t recognise ITP as a legitimate role for them, although GPs were more likely to feel this role was accepted (33.1% of Nurse Prescribers and pharmacists compared with 52.6% of GPs stated that ITP was seen as a legitimate role by their employers). Given the high percentage of participants reporting doing ITC at least some of the time, these data suggest that ITP is an aspect of practice that may be happening ‘under the radar’. Free text comments echoed these responses with reported lack of support from both non-front line staff, the organisational systems (including quality structures and pay for performance) designed to drive care, and patients.

“[ITC needs] recognition from the powers that be that this is a good thing” (GP)

“patients need to understand not prescribing as important as prescribing” (Pharmacist)

In summary, survey findings revealed that, in principle, ITP is recognised as an integral (valued and valuable) part of professional practice but its lacks a clear and consistent account of what it is and why it matters. Furthermore, its value is not perceived to be recognised by the wider healthcare community.

### Engagement

NPT states that for ITP to be integrated into usual care, key individuals must lead the work whilst others must be willing to both get involved and then stay involved in this form of work – including operationalising the tasks in the context of frontline care [17].

As previously stated, some people described themselves as doing ITP; more wished to be more involved. Free text responses offer insights into perceived enablers and barriers to engagement, with four themes identified (Tables 3 and 4): leadership, levels of engagement, patient engagement and barriers to engagement.

Leadership for this work comes from individuals, but is also supported by collective action in the form of multidisciplinary team working (Table 4). Different levels of engagement were evident. Some professionals supported the idea in principle but did not see it as part of their own role to deliver this form of care. Others wanted to engage more with at least some aspects of practice, for example the discontinuation of medicines in vulnerable people. Patient support for this form of practice was acknowledged as an important enabler for professional engagement. Factors external to the health service, including the media, were recognised as key to mobilising patient support.

“it can be difficult to persuade patients and carers to change medicines they’ve been using for a long time” (Pharmacist)

Key emerging barriers to engagement related to the practical constraints of workload placing limits on the necessary time and ‘head space’ needed to engage with this complex form of clinical practice.

“limited by time, caseload and so lack of mental capacity” (GP)

“I barely get through the day reacting” (GP)

The survey findings again indicate a variable response in this second domain of work described by NPT. Results highlighted professionals both leading and engaging with work to deliver ITP, but also demonstrated significant barriers to engagement, notably in prioritising this work within a busy wider schedule of service delivery. The importance of engaging patients, as a key resource to support this activity, was highlighted.

### Action

The third domain of work described by NPT focuses on action – that the people tasked with delivering ITP are able to do the tasks required of them. This includes the need for specific activities of work to be allocated to those with the right skills to do them, the provision of necessary resources by the host organisation, and that people then do the work [17]. Again, the data reveal a varied picture within five key themes: formal training, experiential learning, collective action, partial action and barriers to action (Tables 3 and 4).

Respondents were asked to indicate if they had ever had formal or informal training in the skills needed to tailor prescribing to individual needs. Table 5 summarises the results which showed low levels of formal training in this form of practice with high proportions of respondents across all professional groups expressing a desire for more training.

**Table 5:** Reported skills, training and support for ITP across professional groups

	Nurse prescribers (*n* = 234)	Pharmacists (*n* = 88)	GP (*n* = 97)	Total (*n* = 419)
Numbers (%) reporting medium or high levels of practice skills in…				
Assessing patient management of medicines	153 (65.4)	66 (75)	75 (77.3)	309 (73.7)
Eliciting patient goals	119 (50.1)	48 (54.5)	57 (58.8)	237 (56.6)
Deciding medicines meds to change	111 (47.4)	55 (62.5)	72 (74.2)	250 (59.7)
Monitoring impact of change	122 (52.1)	39 (44.3)	50 (51.5)	226 (53.9)
Described support from [n(%)]				
My training	114 (48.7)	47 (53.4)	43 (44.3)	219 (52.3)
My professional status	80 (34.2)	38 (43.2)	48 (49.5)	176 (42.0)
My colleagues	131 (60.0)	57 (64.8)	61 (62.9)	264 (63.0)
My patients	130 (55.6)	53 (60.2)	73 (75.3)	272 (65.0)
Expressed training experience/needs [n(%)]				
Had formal training	39 (16.7)	16 (18.2)	12 (12.4)	74 (17.7)
Had informal training	81 (34.6)	37 (42.0)	51 (52.6)	178 (42.5)
Would like more training	129 (55.1)	59 (67.0)	67 (69.1)	267 (63.7)

However, people described having informally acquired skills, described as “experience gained intuition” gathered through “practice…over a long time” (Table 4). Practitioners identified both patients and colleagues as important sources of support for this form of practice – more so than formal training or professional status (Table 5). Free text responses revealed the significance of collective action (multidisciplinary working and discussions) in supporting this complex form of practice (Tables 3 and 4). The importance of peer discussion to support this form of practice was highlighted. This was accessed through a number of routes, including multidisciplinary team meetings, learning from trusted colleagues and influential figures, and the value of peer discussion including on-line discussion groups.

We observed professional differences within reported levels of confidence in different aspects of the role of ITP (Table 5), reflecting the level of prescribing-related training and experience within each group. Pharmacists and GPs were generally (although not consistently) more confident in deciding on medicines to change. Nurses and GPs were more comfortable with monitoring the impact of changes – perhaps reflecting their continuing clinical contact with the patients. Overall, there was greater expressed confidence in aspects of prescribing practice that also form part of the usual practice of medicines management – namely assessing patients’ own management of their medicines. A relatively low proportion of respondents expressed confidence in eliciting patient goals for their medication, a finding supported by the existing literature.

Consistent with this mixed picture with reference to training and experience, participants also described ‘partial action’ – an incomplete tailoring of prescribing. For example, they described finding it easier to stop medicines than to think differently about starting them. They called for more practical resources to help them translate an ideal of practice into reality on the ground (training, a framework of practice, practical advice).

“This was not taught when I trained. A guide to implement this safely would be helpful” (GP)

Drawing on past research, we asked participants to describe whether they had experienced previously described [15] barriers to individual tailoring of care. Professionals report high levels of these known barriers (Table 6). Over half reported time as a significant barrier, 43.9% reported competing pressures and 40.5% reported making/recording a defendable decision as a key barrier to ITP. Interestingly about half of GPs compared with one third of nurse prescribers described this latter barrier. Potentially linked with this was concern about a lack of resources to support estimation of risk – see Table 4).

**Table 6:** Number (%) of respondents reporting previously identified barriers to ITP

Barriers:	Time	Competing pressures	Risk stratification	Defend decision	Lack risk estimation support
Nurse prescriber (*n* = 234)	91 (38.9)	76 (32.5)	42 (17.9)	87 (37.1)	83 (35.5)
Pharmacist (*n* = 88)	48 (54.5)	45 (51.1)	31 (35.2)	38 (43.2)	39 (44.3)
GP (*n* = 97)	87 (89.7)	65 (67)	45 (46.4)	49 (50.5)	68 (70.1)
All (*n* = 419)	235 (56.1)	195 (46.5)	120 (28.6)	180 (43.0)	200 (47.7)

Organisational structures including performance monitoring were also consistently highlighted as a barrier to this way of working. Respondents described that they lacked the necessary resources from their host organisation to support this work, although with interprofessional differences in perceptions. Only 118 (50.4%) of nurse prescribers and 45 (51.1%) of pharmacists described having sufficient resources, whilst 72 (74.2%) of GPs identified that that they did have sufficient resource. It is unclear whether this represents a true difference in the support offered to professional groups or an artefact of the research process with variable understanding of what the survey instrument meant by ‘resource’.

Perhaps the most stark identified barrier was the theme of ‘fear’ – of being castigated by other professionals for not following guidelines, of the medicolegal implications of this way of working, and a lack of confidence in making and recording “defendable decisions”.

“a fear of making a mistake and the potential consequences”(Nurse Prescriber)

“hard to record decision and be sure will be understood” (GP)

There was a call for additional qualified and experienced staff to support colleagues in this form of practice. The need for continuity of senior support, in terms of both clinical mentorship and medicolegal advice, was highlighted.

In summary, responses in the Action domain revealed the practical challenges in delivering a model of care that many supported in principle. Much of the existing training for this role was identified as experiential, including recognising the importance of peer support from colleagues. There was suggestion from some, but not all, that formal GP training may support this way of working. The recurring theme of lack of time and energy once more appeared strongly – of exhausted staff being unable to perform this highest-level clinical function. The other key identified barrier was fear – of not being supported in this form of decision making by other health professionals, by the ‘system’ and perhaps by patients too. This is an issue that feeds in to our final NPT domain.

### Monitoring

NTP states that for ITP to be integrated into usual practice, stakeholders must get feedback about the effect of the intervention; individuals and the collective must assess the feedback as being supportive of the tasks of ITP; and feedback must support ongoing learning and development [17]. Analysis in this domain again revealed significant concerns/barriers to integration.

In an era of evidence-based, or evidence-informed, medicine, we asked our participants if they were aware of any evidence supporting ITP. Fifty-five respondents (13.1%) said yes; 290 (69.2%) said no.

Only one in four (26.3%) participants described having received feedback on their ITP, and of these, 37.2% describing that feedback had changed their practice. There was no difference identified between professional groups. Free text responses revealed four themes (Tables 3 and 4): mixed feedback, challenges of feeding back, challenging the status quo, and potential power of feedback.

Feedback had a mixed effect on practice. Positive feedback helped build confidence, encouraging staff to “prescribe less not more” and in a more “patient-centred” approach. One Nurse Prescriber described “each time I see a positive effect, I am motivated to do more”. However, others spoke of how complaints from both colleagues (in secondary care) and carers made them warier:

“I stopped metformin in a 90-year-old with dementia, daughter complained, made me wary to deprescribe” (GP)

Others also reported current feedback mechanisms (performance monitoring) as a significant barrier – the “pressure to prescribe to QOF” (GP) with only “small deviations” permitted.

Participants reflected on the challenges of demonstrating the impact of individually tailored care beyond the effect on an individual patient – in recognising potential benefits.

“will be difficult to quantify downstream costs [that] will be saved - mainly by those outside of my practice so i will do work and savings will be made elsewhere” (GP)

But recognised the potential power of feedback as a source of support for practice commenting that individually tailored prescribing should be a “key performance indicator” (GP).

In summary, responses revealed that current feedback–whether formal or informal – is largely non-supportive or even unsupportive of ITC. Performance monitoring therefore functions as a further barrier to changing to an individually tailored model of prescribing. Respondents revealed that positive/reinforcing feedback comes mainly from patients as well as discussion with colleagues – through informal sources.

## Discussion

### Summary

Our findings revealed general support for a principle of ITP but significant variation and inconsistency in understanding and implementation of practice. Key themes emerging from our analysis describe 3 *enablers* and 4 *barriers* for ITP (Table 7). ITP is recognised as an integral part of professional practice but with a *lack of clarity* in describing what it is which limits wider engagement and understanding. ITP is *not prioritised* within current models and organisation of care. Delivery of ITP is supported by experiential and peer learning but professionals identify a need for more help in developing the *practical skills to support Defendable Decision Making*. Professionals highlight the importance of being able to follow up patients in order to monitor the impact of decision making, but also the need to review the *impact of formal performance monitoring* as a barrier to ITP. From these findings, we describe four key implications for practice: the need to raise understanding of ITP as a legitimate part of professional practice; to prioritise the work of ITP within the range of individual professional activity; to improve the consistency of training and support for interpretive practice; and to review the impact of formal and informal monitoring processes on practice.

**Table 7:** Summarising key emerging themes related to Individually Tailored Prescribing

NPT Domain	Emerging themes	Identified Enablers and barriers	Implications for practice
Sense making	ITP is valued ITP is valuable ITP lacks clarity Value is not recognised/shared	ITP is an INTEGRAL part of professional person-centred practice, But LACKS CLARITY amongst professionals, patients and the wider community	Need work to raise UNDERSTANDING of ITP as a legitimate part of the expert generalist clinical role
Engagement	Leadership Levels of engagement Patient engagement Barriers to engagement	Professionals lack the time, energy and head space to be engaged with this way of working as ITP is NOT PRIORITISED within current models of practice	Need work to PRIORITISE ITP within the range of services within primary care
Action	Formal training Experiential learning Collective action Partial action Barriers	Much of the support and training for ITP comes from experiential learning and peer support. Particular areas of concern for practitioners are in making and recording DEFENDABLE DECISIONS; and getting PRACTICAL ADVICE on how to translate ideals of professional practice in to care on the ground	Need TRAINING and SUPPORT for INTERPRETIVE PRACTICE
Monitoring	Mixed feedback Challenges of feeding back Challenging the status quo Potential power of feedback	The importance of feedback from/learning from patients to support ITP emphasises the significance of CONTINUITY of care. The need for formal monitoring/ feedback to RECOGNISE this complex form of practice	Need to support informal feedback and monitoring through peer reflection and continuity with patients; and consider the impact of formal monitoring on care

### Comparison with existing literature

The identified barriers resonate with, and extend, the findings of our previous survey on barriers to generalist practice [15]. The current survey recognises that clarity of understanding, prioritisation of work, training and feedback remain issues that need to be addressed in the new model of care. However, the survey adds to previous findings by highlighting the importance of patient understanding of the model of care as a potential enabler and barrier. Patient support was identified as a key driver for ITP by respondents; fear of patient dissent or complaint as a barrier. This survey extends the previous findings in noting the potential for multiprofessional groups in supporting engagement with, and so delivery of this model of practice. Although all professional groups recognised the need for more training, pharmacists in particular were identified as an underdeveloped resource. The importance of medicolegal training and support was a strong theme, and resonates with informal discussions on this topic that we have held with professional stakeholder groups. The revised PRIME Prescribing model will recognise the need for medicolegal training in its training programme. Finally, the results highlight the importance of shared reflection and monitoring and of peer professional support, an extension of our previous survey [15]. This observation also resonates with the wider literature on professional practice. Gabbay and Le May describe the importance of peer reflection in supporting complex, beyond guideline practice [19].

Our survey also resonates and extends observations from previous qualitative work. Both Denford and colleagues [2] and Sinnott [20] highlight professional variation in understanding of the process of ITP but also recognise the significance of a process of compromise. Our survey extends those observations to a larger sample, and across professional boundaries. Barriers of time and resource to engagement with ITP have been previously described [586 but our findings go further in emphasising the importance of ‘head space’. The need is not simply for more time, but for protected time that recognises the significant intellectual challenges of this work. Practitioners may not just need more time, but fewer other demands/activities to leave them with sufficient mental capacity for the work involved.

The RCGP recognises GPs as expert generalists [21]. Our survey findings suggest that many are working in that role – able to individually tailor care to the needs of a whole person, the individual. However, participants also report a significant gap in their training – a lack of formal training to deliver these tasks. Indeed, many report training being acquired through experience and learning from significant others. This requires us to have a workforce able to act in that mentor role, and with people working in roles and rotas that allow them access to that apprenticeship. The findings highlight a need to look again at the RCGP training curriculum to both strengthen formal training and protect the informal.

Concerns about the impact of current performance monitoring on individually tailored practice have been previously recognised [20]. Our survey offers new data in highlighting professional suggestions that monitoring could be explicitly used to encourage this model of care [21].

### Strengths and limitations of our work

Our work describes theory informed research, underpinned by the principles described in the MRC Complex Interventions Framework [22] to develop a new complex intervention. Support for our emerging model comes from the observation that our findings resonate with previous research and with the experiences of clinical audiences we have shared the findings with. This report offers a transparent description of development of an intervention supported by literature and empirical findings.

However, we recognise a number of ongoing limitations with the work. This survey focused only on professional perceptions. We have public and patient partners actively working with us on the development of all stages of this work [23], and we have actively prioritised literature on patient experiences of medicines use in designing our work [24]. However, we still need to more formally examine the views of patients who have been unconnected with the work to date.

The mixed responses reveal some lack of clarity/understanding of what we meant by ITP in our survey which requires us to exercise caution in interpreting and extrapolating our findings. We deliberately chose not to fully describe the proposed PRIME Prescribing model in our survey so as to allow people to comment on their own current practice. But our work may now benefit from repeating the study with a clearer description of the specific intervention that we are proposing in the participant information.

Our snowball sample was never intended to be fully representative. Our research methodology is based on a translational approach – action research involving clinicians and academics working together to develop implement and evaluate new models of working [25]. As such the current model is evidence and stakeholder informed, but subject to ongoing review through implementation and formative evaluation.

### Implications for research and practice

The findings from this study are being used to refine the description of our PRIME Prescribing intervention*.* Key additions include the inclusion of a section on medicolegal issues and patient safety within the educational component supporting interpretive practice [26] and clinical decision making. We will work with our patient partners to describe practical support for clinicians in engaging their patients in a change to ITP, and facilitate proactive peer reflection and support to enhance the critical professional learning needed to support ‘beyond protocol’ care [19]. We will actively work with stakeholders to consider how we might include a key performance indicator based on fidelity of delivery of a process of individually tailored care. Finally, we will be actively working to raise the profile and understanding of ITP amongst patient and non-clinical groups (for example, service managers).

## Conclusion

The 2013 Kings Fund report argued that to optimise the potential benefit of polypharmacy on patient-centred outcomes, we must find ways to support compromise between the views of clinicians and patients when making decisions about medicines use [1]. Our survey findings highlight that such work is already happening, but inconsistently and with insufficient support. We have described professional perceptions of changes needed to enhance the individually tailored use of medicines. We now propose to test the findings of this study through incorporating them into the refined description of the complex intervention that is PRIME Prescribing, and so evaluate the impact of introducing these changes to practice.

## Additional file

Additional file 1: FLIPMEDS: the survey tool. Details the survey tool used in the study. (DOCX 20 kb)

## Abbreviations

EGP: Expert Generalist Practice
ITP: Individually Tailored Prescribing
MRC: Medical Research Council
NPT: Normalisation Process Theory
RCGP: Royal College of General Practitioners
SAPC: Society for Academic Primary Care
