# Supplementary material for: Identifying enablers and barriers to individually tailored prescribing: a survey of healthcare professionals in the UK
Source: BMC Fam Pract. 2018 Jan 15;19:17. doi: 10.1186/s12875-017-0705-2 (PMC5769369; doi:10.1186/s12875-017-0705-2)
Supplement: Additional file 1: — FLIPMEDS: the survey tool. Details the survey tool used in the study. (DOCX 20 kb) [file 12875_2017_705_MOESM1_ESM.docx]

**Additional File 1: The Survey instrument**

**Tackling problematic polypharmacy: managing medicines differently**

**An invitation to take part in a survey of medicines management**

***We need to think differently about how we use and manage medicines***

Medicines are an important part of modern health care. Medicines can make people feel better, function better, and live longer. More people than ever now use several medicines at a time (polypharmacy) and over a long period to improve their health.

But we are now seeing a new problem emerging – that of problematic polypharmacy. When the potential benefits of using multiple medicines is outweighed by the burden on patients associated with their use.

There is a call to better tailor how we use medicines to fit with the needs of an individual in order to avoid these problems. But when clinicians tailor medicines to an individual patient, they may need to deviate from the recognised route of ‘best practice’ described, for example, in guidelines.

And practitioners have told us that can be difficult to do.

***We are working with patients, doctors, pharmacists, managers and researchers to develop a new way of supporting individual tailored use of medicines***

We have been developing a new model of care (FLIPMEDS) which supports health practitioners and health systems in delivering individually tailored prescribing of medicines.

Our approach is built upon the expertise of generalist decision making. And supports the clinician in doing three tasks:

- Assessing individual burden of illness and care
- Identifying health-related modifiable points of change
- Designing, recording and implementing an individually tailored plan for medicines use

***To make sure our research helps us all to use medicines in the best way for our patients, we need to hear from you about our ideas.***

We are preparing a bid to the National Institute for Health Research for funding to support this work. Your feedback would be invaluable in helping us make sure our proposal generates research that will make a difference to current practice.

We invite you to send us comments on our ideas by filling in this brief survey. It should take no more than ten minutes of your time. The survey is entirely anonymous.

We will analyse the data from the survey and use it to inform the bid we are developing. We also plan to write up the findings in a report which we aim to publish in an academic journal.

If we are successful with our bid, we will also be looking for people to take part in our research study. We will tell you at the end of the survey how you can find out more about getting involved in this part of the work.

If you have any questions or would like to speak with us before, or after, completing the survey, please contact Joanne ([j.reeve.1@warwick.ac.uk](mailto:j.reeve.1@warwick.ac.uk))

You are free to leave the survey at any time and your data will not be saved.

*Some brief information about you to start with*

What is your main clinical role:

Doctor (GP, primary care), Doctor (Secondary care), Nurse (with prescribing rights)

Nurse (without prescribing rights), Pharmacist, Other (please state)

How old are you?: 20- yrs, 30-, 40-, 50-, 60-, 70-, prefer not to say

Which region do you work in: England NW, NE, Midlands, SW, SE; Scotalnd, Wales, Ireland, Other

Would you describe yourself as Male Female

FLIPMEDS is a new model of practice to support Individually Tailored Prescribing of medicines.

It recognises that when clinicians tailor medicines to an individual patient, they may need to deviate from the recognised route of best practice described, for example, in guidelines.

**These questions assess whether people see Individually Tailored Prescribing (ITP) as a distinct and valuable intervention (Sense Making)**

1. Do you feel you currently provide individually tailored medicines use for your patients?

YES, always Yes, sometimes, NO

*Please explain your answer (free comment)*

Would you like to provide more individually tailored prescribing of medicines for your patients?

YES, NO

1. Do you feel other colleagues you work with are providing individually tailored prescribing for patients? YES, always YES, sometimes, NO

**These questions assess if participants are or would potentially Engage with ITP**

1. Do you feel that providing individually tailored prescribing, including - where necessary - beyond guideline prescribing, is a legitimate part of your role?

YES, NO

Comments (free text)

1. Do you feel that the organisation within which you work (be that a GP practice, pharmacy hospital) regards providing individually tailored prescribing, including - where necessary - beyond guideline prescribing, is a legitimate part of your role?

YES, NO

Comments (free text)

1. Individually Tailored Prescribing is a resource intensive way of working. Do you think that the NHS should prioirtiise resources to support practitioners to work more in this way?

YES MAYBE NO

Comments (free text)

**These questions ask if people have the skills and resources to undertake the work (Action)**

1. Have you ever had any FORMAL training in making individually tailored decisions about medicines use, including decisions which go beyond a guideline

YES NO

1. Have you ever had any INFORMAL training in making individually tailored decisions about medicines use, including decisions which go beyond a guideline

YES NO

1. Please describe any training that you have had
2. Are there any particular elements of individually tailored prescribing that you feel more confident doing? (Tick all that apply)

Finding out from patients how they are managing their medicines on a daily basis

Finding out from patients how they make decisions about using their medicines (eliciting their priorities and goals)

Deciding which medicines could be changed (reduced/switched)

Monitoring the impact of any change

Other (please specify)

1. Do you think additional training and support could help you deliver more individually tailored care for your patients?

YES NOT SURE NO

Comments (free text)

1. Do you have sufficient resources to provide individually tailored prescribing for your patients?

YES NOT SURE NO

Comments (free text)

1. People have previously suggested a number of things that help them deliver individually tailored prescribing to their patients. Do you agree?

*Tick all that apply*

Training YES NO

My professional status YES NO

Support of colleagues YES NO

Patients YES NO

Others (please state)

1. What, if any, barriers do you experience in delivering individually tailored prescribing of medicines to patients?

Comments

1. People have previously suggested a number of barriers. Please tell us which, if any, of these stop you from delivering individually tailored prescribing

Lack of time in the consultation YES NO

Too many other pressures in the working week YES NO

Accessing the patients who most need this approach YES NO

Lack of training in balancing multiple uncertainties and creating defendable decisions YES NO

Lack of resources in estimating the risk and benefit of medicines for individuals YES NO

**These questions ask if people have had feedback that supports or inhibits ITP (Monitoring)**

1. Are you aware of any evidence, audit data or other information about the impact of individually tailored prescribing? YES NO
2. Have you had any feedback about your own Individually Tailored Prescribing? YES NO N/A

If yes, please describe

1. Has feedback from your past decisions about using medicines changed the way you practice?

YES NO

IF YES, HOW

1. How could feedback on Individually Tailored Prescribing help improve your practice?

Free text

Thank you for your time in completing this survey.

If you have questions contact j.reeve.1@warwick.ac.uk

If you would be interested in taking part in our research, click here to take you to a new page to find out more.
